# Clinical Course of Laryngeal Granuloma Without Surgical Treatment

**DOI:** 10.1155/DTE.7.129

**Published:** 2001

**Authors:** Satoshi Horiguchi, Mamoru Suzuki, Hideo Takagi, Toshiro Yamanishi, Kazuhiro Nakamura

**Affiliations:** Department of Otolaryngology Tokyo Medical University 6-7-1 Nishi-shinjuku Shinjuku-ku Tokyo 160-0023 Japan

## Abstract

Laryngeal granuloma is a rather common pathological entity, but its therapeutic strategy is still controversial. In general, therapeutic strategy consists of medications such as steroids or tranilast, in addition to vocal hygiene and surgery. Surgical removal is most commonly performed. However, it has recently been reported that recurrence after surgery is high.

We successfully treated 19 of 20 cases of laryngeal granuloma without surgical removal. It took 28–328 days for the granulomas to disappear. Therefore conservative treatments should be the first choice of treatment.


*Diagnostic and Therapeutic Endoscopy*, Vol. 7, pp. 129–133
Reprints available directly from the publisher
Photocopying permitted by license only

© 2001 OPA (Overseas Publishers Association) N.V.
Published by license under
the Harwood Academic Publishers imprint,
part of Gordon and Breach Publishing
a member of the Taylor & Francis Group.
All rights reserved.

### Clinical Course of Laryngeal Granuloma Without Surgical Treatment

SATOSHI HORIGUCHI*, MAMORU SUZUKI, HIDEO TAKAGI, TOSHIRO YAMANISHI and KAZUHIRO NAKAMURA

*Department of Otolaryngology, Tokyo Medical University, 6-7-1 Nishi-shinjuku, Shinjuku-ku, Tokyo 160-0023, Japan*

*(Received 6 June 2001; In final form 10 August 2001)*

Laryngeal granuloma is a rather common pathological entity, but its therapeutic strategy is still controversial. In general, therapeutic strategy consists of medications such as steroids or tranilast, in addition to vocal hygiene and surgery. Surgical removal is most commonly performed. However, it has recently been reported that recurrence after surgery is high.

We successfully treated 19 of 20 cases of laryngeal granuloma without surgical removal. It took 28–328 days for the granulomas to disappear. Therefore conservative treatments should be the first choice of treatment.

*Keywords*: Clinical course; Conservative therapy; Granuloma; Larynx

### INTRODUCTION

Non-specific granuloma of the larynx was first reported as "contact ulcer of the larynx" by Jackson [1] in 1928. In 1932, Clausen [2] reported laryngeal granuloma as an unusual sequela of tracheal intubation. Since then, laryngeal granuloma has been thought to be a rather common pathological entity. However, its therapeutic strategy is still controversial. The etiology of laryngeal granuloma varies. Inappropriate vocal use, habitual coughing, and endotracheal intubation are well-known causes. Recently, gastroesophageal reflux has attracted attention as a cause.

In general, therapeutic strategy consists of medication such as steroids or tranilast, in addition to vocal hygiene and/or surgery. Surgical removal is most commonly performed. However recently, conservative therapy has been given priority, since the recurrence rate after surgical removal is high. Causes, treatments and outcome of laryngeal granuloma are summarized in Table I [3–6].

The main purpose of this study was to observe the changes in shape and size of laryngeal granuloma

*Corresponding author. Tel. +81-3-3342-6111. Ext. 5788. Fax: +81-3-3346-9275

TABLE I Cause, treatments and outcome of laryngeal granuloma

Cause
- Endotracheal intubation
- Idiopathic (chronic cough, vocal abuse, foreign body, gastroesophageal reflux, etc.)

Treatments
- Surgical (microscopic endolaryngeal with/without laser)
- Pharmacological (steroid, tranilast, proton pump inhibitor, botulinum toxin, etc.)
- Conservative (vocal hygiene, voice training, etc.)

Outcome
- Cure
- Therapy-resistant
- Tendency to recur treated by conservative treatment without surgical removal.

### SUBJECTS

From April 1997 to April 1999, 20 patients with laryngeal granuloma were treated conservatively at the Department of Otolaryngology, Tokyo Medical University Hospital. The patients were from 24 to 69 years old, and consisted of 14 men and 6 women. Five cases had a history of vocal abuse and another five had endotracheal intubation. Two coughed frequently. The remaining eight had no recognizable cause. None of them complained of any symptoms suggesting gastroesophageal reflux.

We instructed them to avoid unnecessary cough and vocal abuse. Steroid inhalants, tranilast and/or proton pump inhibitor were given for some patients during the follow-up period. No surgical procedure was performed on any patient. (See Table II).

TABLE II Subjects

| | | |
|---|---|---|
| Cases | Total 20 cases (14 men and 6 women, age 24–69 y.o.) | |
| Cause | After intubation | 5 cases |
| | Vocal abuse | 5 cases |
| | Habitual cough | 2 cases |
| | Unknown | 8 cases |
| Treatment | Conservative | |
| | ① Vocal hygiene and Voice training | |
| | ② Additional medication (steroid inhaler, tranilast, proton pump inhibitor) | |

During follow-up, ruling out the possibility of malignancy is very important. We carefully watched and performed biopsy whenever necessary, however, no case aroused any suspicion of malignancy.

### RESULTS

Figure 1 shows a case of bilateral granuloma after endotracheal intubation. The patient was a 26-year-old woman. She had received a mandible operation for malocclusion under general anesthesia on April 15th, 1997. Since then, she sometimes felt difficulty on phonation. She visited an otolaryngologist and abnormal masses were found in her larynx. She was referred to our clinic on June 16th, 1997 for further examination. Day 0 indicates the day of the first visit to our clinic and start of the treatment. Bilateral laryngeal granulomas were observed on both vocal processes. She had no other history connected to laryngeal granuloma, apart from endotracheal intubation. On day 30, the granuloma on the left disappeared and the other on the right changed its shape and became pedunculated. Finally, on day 120, the latter granuloma disappeared. The patient noticed bloody sputum on day 100. We speculate that the granuloma was possibly exfoliated on that occasion.

Figure 2 shows a case of idiopathic granuloma. The patient was a 52-year-old man. He had felt a foreign body sensation in the throat since July 1997. On August 20th, 1997, his otolaryngologist found a small polypoid lesion, which showed a tendency to grow, on his left vocal cord. On September 27th, 1997, he was

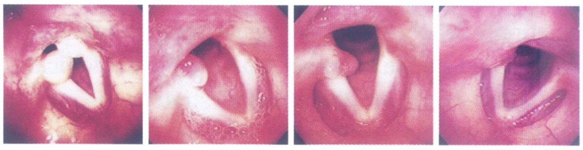

**Day 0 Day 30 Day 81 Day 120**

**26-year-old woman (after intubation)**
**The left granuloma shrank and disappeared.**
**The right granuloma became pedunculated, then, was thought to have been exfoliated.**

FIGURE 1 Laryngeal granuloma after intubation.

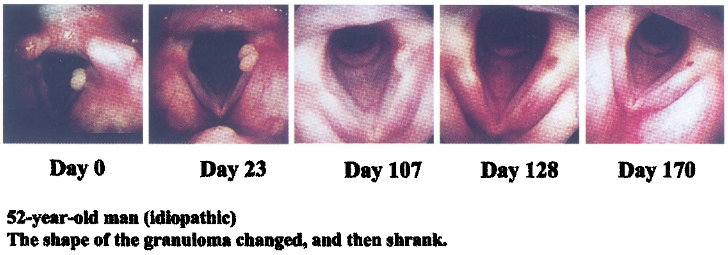

FIGURE 2 Idiopathic laryngeal granuloma.

referred to our clinic for further examination. Day 0 indicates the day of the first visit to our clinic. A bunchy granuloma was present on his left vocal process. On day 23, the granuloma changed in shape. On day 79, it shrank, and on day 128, it disappeared. On day 170, the mucosal surface of the left vocal process looked normal, although there was submucosal pigmentation.

Another small granuloma was found on his right vocal process on June 3rd, 1998, but it also disappeared by October 21st, 1998.

Figure 3 shows a recurrent granuloma after surgical removal. The patient was a 63-year-old man. He had no complaint related to his throat or voice. A laryngeal granuloma was found during gastroscopy in a regular health check-up on November 7th, 1996. The granuloma was excised using a KTP laser under microscopic endolaryngeal surgery. Day-272 is the day just before surgical removal was done at another hospital. However, a recurrence of granuloma was found during regular health check-ups although he had no symptoms. On June 11th, 1997, he was referred to our clinic for conservative treatment. Again, day 0 is the day of his first visit to our clinic. We taught him vocal hygiene, avoidance of unnecessary coughing and prescribed steroid inhalation. On day 28, the granuloma decreased in its size, and from day 61, it was seen no more.

Nineteen out of 20 patients with laryngeal granuloma were cured without any surgical intervention. It took from a minimum of 28 days to a maximum of 328 days before they disappeared. A small granuloma recurred on the contralateral vocal process in three cases, however, all of them disappeared in 3 months. No ipsilateral recurrence was observed in our cases.

The remaining single case is still receiving regular follow-up. In this particular case, the patient could not avoid habitual occupational vocal abuse.

### DISCUSSION

Peacher and Hollinger [7] first emphasized the importance of vocal therapy for laryngeal granuloma in 1947. However, the recurrence rate, average healing time and recurrence-free period depend on the

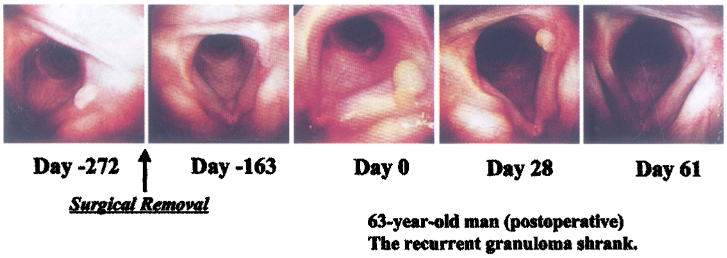

FIGURE 3 Recurrent laryngeal granuloma.

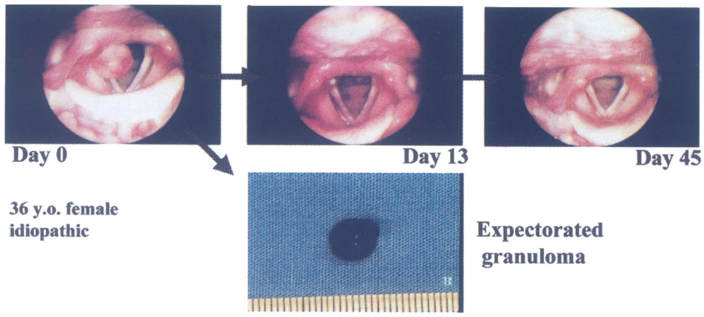

**from S.Horiguchi, I.Kawabata: Spontaneous expectoration of laryngeal granuloma. J.Jpn.Bronchoesophagol.Soc., 40(6), 1989. (Reprinted with permission)**

FIGURE 4 Spontaneous expectoration of laryngeal granuloma.

therapeutic modality and differed from one investigator to another.

The benefit of pharmacological therapeutic strategy is still unclear. Anti-reflux treatment is expected to be effective [5,6] only when the granuloma is definitely induced by gastroesophageal reflux. Steroid inhalation seems to be a favorable option, but it is still unknown if it shortens the healing process.

We assume that the clinical course of laryngeal granuloma has two patterns. One is, the granuloma shrinks by itself with time. The other is that granuloma changes into a pedunculated polypoid lesion and is then exfoliated. Figure 4 is from our previous paper entitled "Spontaneous expectoration of laryngeal granuloma" [8]. It is reported that the granuloma can be expectorated out via the mouth following excessive coughing. In our series, 8 of 20 granulomas cleared up in a short term. In these cases, we assume the granuloma was exfoliated.

Figure 5 shows our hypothesis concerning the formation and healing process of granuloma. It is a primarily inflammatory granulomatous mass, caused by the disturbed healing process of ulcer or erosion. The pathological healing process is introduced by several triggers, such as chronic vocal abuse, habitual cough and endotracheal intubation [9,10].

Even if granulomatous tissue is completely removed under microscopic surgery, without avoidance of these triggers, recurrence is common.

We hypothesize that, if a patient can avoid these triggers, the granulomas shrink naturally or are pushed out by normal epithelization around the stem or base. Since granuloma is induced by biomechanical friction on the vocal process, vocal therapy seems to

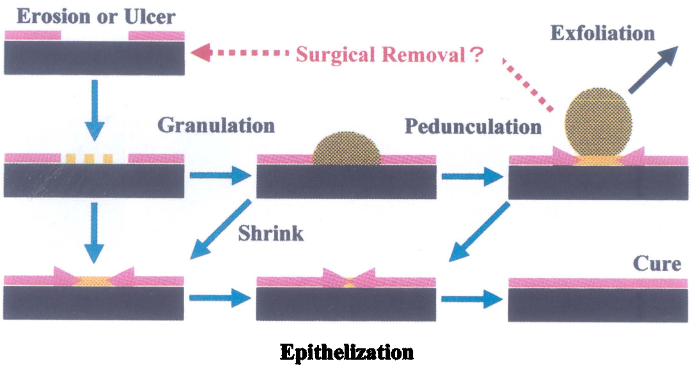

FIGURE 5 Hypothesis of clinical course of laryngeal granuloma.

be most essential not only as the initial therapy but also for prevention of recurrence.

### CONCLUSIONS

1. Nineteen out of 20 laryngeal granulomas were cured without surgery. Therefore conservative treatments should be first considered for this entity.
2. Laryngeal granulomas often change into pedunculated polypoid lesions and then are exfoliated. Even if a granuloma does not shrink initially, it is best to follow up and wait using conservative treatment until its exfoliation. While observing, ruling out the possibility of malignancy is very important. For this purpose, we should not hesitate to do biopsy.
3. If patients fail to avoid the triggering cause, recurrence should be expected. Our hypothesis suggests that, voice therapy is the best adjunct therapy.

### *Acknowledgement*

The authors are indebted to Prof. J. Patrick Barron of the International Medical Communications Center of Tokyo Medical University for his review of this manuscript.

### *References*

[1] Jackson, C. (1928) "Contact ulcer of the larynx", *Ann. Otol.* **37**, 227–230.
[2] Clausen, R.J. (1932) "Unusual sequela of tracheal intubation", *Proc. R. Soc. Med. Part 2* **25**, 1507.
[3] Kawaida, M., Fukuda, H., Kawasaki, Y., *et al.*, (1996) "Conservative therapy for nonspecific granuloma of the larynx using a beclomethasone dipropionate inhaler", *Diagn. Ther. Endoscopy* **3**, 93–98.
[4] Cary, M.A., Sapienza, C.M., Cassisi, N.J. and Moore, G.P. (1998) "A preliminary report on treatment of contact granuloma with steroid injections", *Am. J. Speech Lang. Pathol.* **7**, 92–96.
[5] Ward, P.H., Zwitman, D., Hanson, D. and Berci, G. (1980) "Contact ulcers and granulomas of the larynx: New insights into their etiology as a basis for more rational treatment", *Otolaryngol. Head Neck Surg.* **88**, 262–269.
[6] Nasri, S., Sercarz, J.A., McAlpin, T. and Berke, G.S. (1995) "Treatment of vocal fold granuloma using botulinum toxin type A", *Laryngoscope* **105**, 585–588.
[7] Peacher, G. and Hollinger, P. (1947) "Contact ulcer of the larynx. II: The role of vocal re-education", *Arch. Otolaryngol.* **46**, 617–623.
[8] Horiguchi, S. and Kawabata, I. (1989) "Spontaneous expectoration of laryngeal granuloma", *J. Jpn. Broncho-esophagol. Soc.* **40**(6), 469–473, in Japanese.
[9] Peacher, G.M. (1961) "Vocal therapy for contact ulcer of the larynx: a follow-up of 70 patients", *Laryngoscope* **71**, 37–47.
[10] Ylitalo, R. and Lindestad, P. (2000) "Laryngeal findings in patients with contact granuloma: a long-term follow-up study", *Acta Otolaryngol.* **120**, 655–659.

